# Breeding for robustness: investigating the genotype‐by‐environment interaction and micro‐environmental sensitivity of Genetically Improved Farmed Tilapia (*Oreochromis niloticus*)

**DOI:** 10.1111/age.12680

**Published:** 2018-07-30

**Authors:** S. Agha, W. Mekkawy, N. Ibanez‐Escriche, C. E. Lind, J. Kumar, A. Mandal, J. A. H. Benzie, A. Doeschl‐Wilson

**Affiliations:** ^1^ The Roslin Institute The University of Edinburgh Easter Bush Campus Midlothian EH25 9RG Edinburgh UK; ^2^ Animal Production Department Faculty of Agriculture Ain Shams University Shubra Alkhaima 11241 Cairo Egypt; ^3^ WorldFish Jalan Batu Maung Batu Maung, Bayan Lepas 11960 Penang Malaysia; ^4^ Institute for Animal Science and Technology Universitat Politècnica de València 46022 València Spain; ^5^ Rajiv Gandhi Center for Aquaculture Vijayawada Tamil Nadu India; ^6^ School of Biological Earth and Environmental Sciences University College Cork North Mall Campus Cork Ireland

**Keywords:** aquaculture breeding, genetic heterogeneity of environmental variance, Nile tilapia, resilience

## Abstract

Robustness has become a highly desirable breeding goal in the globalized agricultural market. Both genotype‐by‐environment interaction (G × E) and micro‐environmental sensitivity are important robustness components of aquaculture production, in which breeding stock is often disseminated to different environments. The objectives of this study were (i) to quantify the degree of G × E by assessing the growth performance of Genetically Improved Farmed Tilapia (GIFT) across three countries (Malaysia, India and China) and (ii) to quantify the genetic heterogeneity of environmental variance for body weight at harvest (BW) in GIFT as a measure of micro‐environmental sensitivity. Selection for BW was carried out for 13 generations in Malaysia. Subsets of 60 full‐sib families from Malaysia were sent to China and India after five and nine generations respectively. First, a multi‐trait animal model was used to analyse the BW in different countries as different traits. The results indicate a strong G × E. Second, a genetically structured environmental variance model, implemented using Bayesian inference, was used to analyse micro‐environmental sensitivity of BW in each country. The analysis revealed the presence of genetic heterogeneity of both BW and its environmental variance in all environments. The presence of genetic variation in residual variance of BW implies that the residual variance can be modified by selection. Incorporating both G × E and micro‐environmental sensitivity information may help in selecting robust genotypes with high performance across environments and resilience to environmental fluctuations.

## Introduction

Robustness, ‘the ability to combine a high production potential with resilience to stressors, allowing for unproblematic expression of a high production potential in a wide variety of environmental conditions’ (Knap 2005), has become a highly desirable breeding goal in the globalized agricultural market. Studies have shown that relative performance of the same genotype can vary markedly in different macro‐environments characterized by, for example, different production systems or climatic conditions (Khaw *et al*. 2012; Sae‐Lim *et al*. 2015b). Recent evidence suggests that genotypes can also adapt differently to changes in micro‐environments characterized by small‐scale spatial or temporal environmental perturbations (Mulder *et al*. 2013). Thus, environmental sensitivity to changes in either the macro‐ and micro‐environment are two important components of robustness (Strandberg *et al*. 2013).

Genotype‐by‐environment interaction (G × E) is defined as the mean phenotypic changes of a given genotype in different environments (Falconer & Mackay 1996). The response of the genotypes to measurable levels of environmental factors, such as water temperature, nutrition and production environments, is termed ‘macro‐environmental sensitivity’ (Mulder *et al*. 2013). G × E can be used as an indication of the presence of genetic variation in macro‐environmental sensitivity. The presence of G × E may imply that the best genotype in one environment is not the best in other environments, leading to genotype re‐ranking across environments in regards to genetic merit, which could potentially reduce the effectiveness of breeding programmes. Micro‐environmental sensitivity, in contrast, refers to the ability of a genotype to be buffered against local unknown environmental fluctuations in a single environment (Falconer & Mackay 1996). It can be quantified by the magnitude of environmental variance of a specific trait, and the genetic influence on this environmental variance is quantified by the degree of genetic heterogeneity of environmental variance (San Cristobal‐Gaudy *et al*. 1998; Hill & Mulder 2010).

Both, G × E and micro‐environmental sensitivity are important robustness components of aquaculture production, in which breeding stock is disseminated to numerous different environments. Many aquaculture species are grown in open uncontrolled environments such as outdoor ponds or cages. Changing the environmental variables in the production rearing locations to be similar to the nucleus breeding environment may be expensive and impractical. Thus, breeding for robustness could be a desirable breeding goal in aquaculture. Although low G × E maximizes consistency in performance across rearing systems in the same or different countries, low micro‐environmental sensitivity improves uniformity in performance, for example, in body weight at harvest (BW) (Ibanez‐Escriche *et al*. 2008; Janhunen *et al*. 2012). This is of particular importance for tilapia*,* the second largest farmed aquaculture species worldwide.

To improve the performance of tilapia, WorldFish has continued the Genetically Improved Farmed Tilapia (GIFT) strain breeding programme in Malaysia, after its original establishment in the Philippines (Ponzoni *et al*. 2011). Currently, GIFT is disseminated to over 16 countries worldwide. However, only a few of these countries have their own breeding programme. The main challenge facing such a multi‐environment breeding programme is to select fish with high performance across environments and resilience to environmental perturbation, i.e. ‘robust fish’ having low environmental sensitivity across a wide range of environmental conditions. The objectives of this study were (i) to quantify the degree of G × E by assessing the growth performance of GIFT across three different countries (Malaysia, India and China) and (ii) to quantify the genetic heterogeneity of environmental variance for BW in GIFT as a measure of micro‐environmental sensitivity.

## Materials and methods

### Data source and breeding programme management

Data were obtained from the WorldFish GIFT breeding programme. The programme began in late 2001 with the introduction of 63 families, from the sixth generation of the selection programme of the GIFT Foundation International in the Philippines, to the Aquaculture Extension Center, Department of Fisheries, Jitra, Malaysia. Average family size was 35 individuals with an average weight of 10 g. Individuals were reared to an average weight of 250 g. Mating started in 2002 to establish the first generation of the GIFT breeding programme in Malaysia. Selection was based on BW. Two lines, named the selection line and control line, were formed based on high and average estimated breeding values of BW respectively (for more information, see Ponzoni *et al*. 2011; Hamzah *et al*. 2014). Selection was carried out for 13 generations in Malaysia. Representatives of 60 families chosen at random from the fifth and the ninth generations of the selection line of GIFT selection programme in Malaysia were then sent to Wuxi City, China, and the Rajiv Gandhi Centre for Aquaculture, Andhra Pradesh, India respectively, where satellite breeding programmes were established. Selection based on BW was then carried out for three and four generations in China and India respectively. Selection was based on between‐ and within‐family selection; thus descendants from the same founder families were represented in each of the three environments. The total number of records in Malaysia was 46 438, representing 1131 full‐sib families from 13 generations, whereas for China and India the total numbers of records were 7053 representing 221 full‐sib families from three generations and 11 205 representing 216 full‐sib families from four generations respectively (Table 1).

**Table 1:** Descriptive statistics of the raw data of Genetically Improved Farmed Tilapia reared in Malaysia, China and India

	Malaysia	India	China
No. of records	46 438	11 205	7053
No. of generations	13	4	3
No. of families	1131	216	212
Average family size	41	52	32
Average grow‐out period (days)	230	232	344
Average body weight at harvest (BW) (g)	222	313	244
Standard deviation of BW (g)	87.77	93.20	108.67
Coefficient of variation for BW	0.40	0.30	0.45

### Statistical analysis

First, a multi‐trait animal model was used for the genetic analysis of the G × E by analysing the BW in different countries as different traits. Second, a single trait genetically structured environmental variance model, implemented using Bayesian inference, was used to analyse micro‐environmental sensitivity of BW in each country.

### Genetic analysis for G × E

Data records and pedigree information from the three environments—Malaysia, India and China—were combined. The full pedigree of the GIFT in the three environments consisted of 64 696 individuals representing 1568 full‐sib families. To meet the normal distribution assumptions of the linear models used in data analysis, BW values were transformed to a square root. Due to differences in rearing conditions in each environment, the fixed effects models across the three countries were as follows:

(1) $$\text{In Malaysia}:y_{\mathrm{ijkl}}=\mu +R_{i}+{\left(S*SP*L\right)}_{j}+\beta_{l}*{\text{AGE}}_{\mathrm{ijk}}{\left(\text{SP}*L\right)}_{l}+e_{\mathrm{ijkl}},$$

(2) $$\text{In China}:y_{\mathrm{ijk}}=\mu +R_{i}+{\left(S*\text{SP}\right)}_{j}+\beta *{\text{AGE}}_{\mathrm{ijk}}+e_{\mathrm{ijk}}\;\text{and}$$

(3) $$\text{In India}:y_{\mathrm{ijk}}=\mu +R_{i}+{\left(S*\text{SP}\right)}_{j}+\beta *{\text{AGE}}_{\mathrm{ijk}}+e_{\mathrm{ijk}},$$

where *y* is the square root of BW; *μ* is the population mean; *S* is the fixed effect of sex (female, male); *SP* is the fixed effect of spawning season (13 levels in Malaysia, three levels in China and four levels in India); *L* is fixed effect of line *j* (control, selection); *R* is the fixed effect of rearing system *i* (cage, pond); S*SP*L is the combined effects of sex, spawning season and line; S*SP_*j*_ is the combined effects of sex and spawning season; *AGE* is the harvest age (nested within spawning season and line in Malaysia) as a linear covariate; and *e* is the residual.

A multi‐trait animal model was used assuming BW in each of the three countries as a different trait to estimate the heritability (*h* ^2^), common environmental effects (environmental effect common to full sibs, i.e. hapa within pond) and genetic correlations using airemlf90 (Misztal *et al*. 2015). The full dataset with full pedigree information for the three environments was used to get unbiased estimates of the variance components and genetic parameters (Henderson 1975). Estimates of the genetic correlations of BWs between environments were used as a measure of the magnitude of G × E (genotype re‐ranking). The mixed model in a matrix notation was:

y=Xb+Za+Wc+e,

where ***y*** is a vector of the square root of the observed phenotypes of BW at three different countries; ***X***,***Z*** and ***W*** are incidence matrices; ***a*** is the additive genetic effect of individual animals; ***c*** is the vector of common environmental full‐sib effects; ***b*** is the vector of fixed effects for each environment as mentioned above and ***e*** is the vector of residuals. The variance–covariance structure can be written as:

$$V\left(\begin{array}{c} a\\ c\\ e \end{array}\right)=\left(\begin{array}{ccc} A⊗G & 0 & 0\\ 0 & I⊗C & 0\\ 0 & 0 & I⊗R \end{array}\right),$$

where ***A*** is the additive genetic relationship matrix; ***G***,***C*** and ***R*** are the additive genetic, common environmental and residual environmental (co)variances matrices respectively, ***I*** is the identity matrix and ⊗ denotes the Kronecker product.

To provide estimates of the genetic correlations between the GIFT from different countries immediately after transferring the fish to India and China, and to monitor their trends over subsequent generations, the analysis of the multi‐trait animal model was repeated on a reduced dataset containing BWs for each generation of both fish in India and China together with the full data of fish in Malaysia.

### Analysis of the genetic heterogeneity of environmental variance

The genetically structured environmental variance model proposed by San Cristobal‐Gaudy *et al*. (1998) and implemented into the Bayesian gsevm‐v.2 software (Ibanez‐Escriche *et al*. 2010) was used to analyse the heterogeneity of environmental variance of the GIFT in each country as a single trait analysis. This model assumes an exponential distribution for the residual variance after systematic (fixed) and common environmental effects and additive genetic effects on the trait mean, BW, have been accounted for and follows the form:

$$y|b,a,c,\sigma_{e}^{2}∼N\left(Xb+Za+Wc,\mathrm{Diag}{\left(\sigma_{\mathrm{ei}}^{2}\right)}_{i=1}^{n}\right),$$

where $\mathrm{Diag}{\left(\sigma_{\mathrm{ei}}^{2}\right)}_{i=1}^{n}$ is the environmental variance diagonal matrix with diagonal entries $\sigma_{\mathrm{ei}}^{2}$ and

$$\ln{\left(\sigma_{\mathrm{ei}}^{2}\right)}_{i}^{n}=X^{*}b^{*}+Z^{*}a^{*}+W^{*}c^{*}.$$

The parameters and matrices related to the environmental variance are denoted with asterisks (*). Vectors ***b*** and b* contain fixed effects in each environment as stated above, and a* is a column vector of additive genetic values affecting environmental variation of body weight. The genetic effects (***a***,a*) were assumed to be multi‐variate normally distributed:

$$\left(\begin{array}{c} a\\ a^{*} \end{array}|G\right)∼N\left(\left(\begin{array}{c} 0\\ 0 \end{array}\right),G⊗A\right),$$

$$G=\left(\begin{array}{cc} \sigma_{a}^{2} & \rho \sigma_{a}\sigma_{a^{*}}\\ \rho \sigma_{a}\sigma_{a^{*}} & \sigma_{a^{*}}^{2} \end{array}\right),$$

where ***G*** is the matrix of additive genetic (co)variances, ***A*** is the additive genetic relationship matrix, the elements of ***G*** are the genetic variances associated with (***a***,a*) and *ρ* is the coefficient of correlation. Vectors ***c*** and c* contain the common environmental effects for full‐sibs in each environment and are assumed to be normally distributed:

$$\left(c|\sigma_{c}^{2}\right)∼N\left(0,I\sigma_{c}^{2}\right)$$

$$\left(c^{*}|\sigma_{c^{*}}^{2}\right)∼N\left(0,I\sigma_{c^{*}}^{2}\right)$$

Details of the *a priori* distributions for vectors ***b*** and b*, the variance–covariance parameters and the Markov chain Monte Carlo (MCMC) implementation to fit the model are described by Sorensen & Waagepetersen (2003). Each MCMC run consisted of 1 000 000 iterations with a burn‐in period of 100 000 iterations. Convergence was tested using the criterion described by Gelman & Rubin (1992). Applying the model to the untransformed observed BWs resulted in lack of convergence, although up to 1 million iterations were used in the analysis. Therefore, to meet the normal distribution assumptions of the linear models used in the analysis and to achieve model convergence, BWs were transformed to square root, in line with the G × E analyses above.

## Results

### G × E

**Figure 1:**
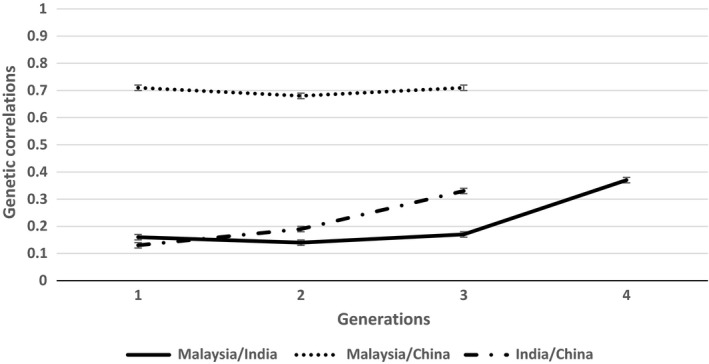
Trend of the genetic correlation and its ± standard error for body weight at harvest for each generation post transfer to either India or China together with the full data for Malaysia.

The descriptive statistics of the raw data of GIFT reared in Malaysia, China and India are shown in Table 1. The average BW of GIFT reared in India was substantially higher for similar or shorter age at harvest than for those reared in Malaysia and China, whereas the coefficient of variation was lower for the GIFT in India than in those in Malaysia and China. The estimates of phenotypic and genetic parameters for growth traits in each production environment, estimated using a multi‐trait animal model, are shown in Table 2. Similar additive genetic variances were observed in the GIFT reared in Malaysia (1.84) and China (1.74), but a lower additive genetic variance was calculated for those reared in India (0.72). The heritability estimates were 0.29 and 0.31 for GIFT reared in Malaysia and China respectively, but lower heritability (0.18) was observed for those reared in India. Genetic correlations for BW for GIFT reared in Malaysia and China were 0.70, whereas genetic correlations for BW for those reared in India and Malaysia and in India and China were 0.37 and 0.33 respectively (Table 3). Overall, low to moderate genetic correlation of BW was found across different environments, which is indicative of the presence of G × E. Furthermore, genetic correlations between GIFT reared in Malaysia and India and those reared in China and India were even lower (i.e. below 0.2) for the first few generations after transferring the fish but increased towards the corresponding estimates for the full dataset over successive generations. In contrast, the estimates for genetic correlation between GIFT reared Malaysia and China were similar to the estimate obtained for the full dataset and remained stable over successive generations (Fig. 1).

**Table 2:** Mean and its standard errors of phenotypic (VP), genetic (VA), common environmental (VC) and residual (VR) variance estimates, heritability (*h* ^2^), common environmental effect (*c* ^2^) and their standard errors (SE) for body weight at harvest of Genetically Improved Farmed Tilapia in each production environment

Environment	VP	VA	VC	VR	*h* ^2^ ± SE	*c* ^2^ ± SE
Malaysia	6.25 ± 0.12	1.84 ± 0.03	2.43 ± 0.12	1.98 ± 0.02	0.29 ± 0.01	0.39 ± 0.01
China	5.60 ± 0.10	1.74 ± 0.03	1.70 ± 0.10	2.62 ± 0.03	0.31 ± 0.01	0.30 ± 0.03
India	4.07 ± 0.21	0.72 ± 0.01	0.73 ± 0.20	2.16 ± 0.04	0.18 ± 0.01	0.18 ± 0.02

**Table 3:** Genetic correlations and (±) standard errors for genotype × environment interaction for body weight at harvest of Genetically Improved Farmed Tilapia

Environment	India	China
Malaysia	0.37 ± 0.01	0.71 ± 0.01
China	0.33 ± 0.01	

### Micro‐environmental sensitivity

Markov chain Monte Carlo estimates of the posterior means and 95% highest posterior intervals for genetic variance for the GIFT reared in Malaysia, China and India, applied to square root transformed body weight, are shown in Table 4. The posterior means of the additive genetic variances of BW at the level of the mean were 0.45, 1.90 and 0.70 for GIFT reared in Malaysia, China and India respectively. A considerable additive genetic variance of BW at the level of the variance ($\sigma_{a^{*}}^{2}$), with the 95% highest posterior interval that did not include zero, was found in GIFT reared in Malaysia (0.34) and China (0.31), whereas a lower estimate (0.12) was observed for those reared in India. The posterior mean of the genetic correlations between the additive genes affecting the mean of transformed BW and its variance (95% highest posterior interval) of GIFT reared in Malaysia and China were −0.53 (−0.47, −0.59) and −0.70 (−0.80, −0.60) respectively. For GIFT reared in India, a lower posterior mean of the genetic correlation (−0.03) with the 95% highest posterior interval that includes zero (−0.17, +0.11) was observed.

**Table 4:** Posterior means (PM) and 95% highest posterior density intervals (HPD95%) of variance components and the genetic additive correlation (*ρ*) between the additive genes affecting the mean body weight at harvest of Genetically Improved Farmed Tilapia and its variance

Variance component	Malaysia			China			India		
	PM	HPD95%		PM	HPD95%		PM	HPD95%	
$\sigma_{a}^{2}$	0.45	0.44	0.46	1.90	2.18	1.62	0.70	0.47	0.93
$\sigma_{c}^{2}$	3.38	3.22	3.55	1.67	1.90	1.44	1.36	1.19	1.53
$\sigma_{a*}^{2}$	0.34	0.31	0.37	0.31	0.39	0.23	0.12	0.09	0.15
$\sigma_{c*}^{2}$	0.14	0.12	0.15	0.122	0.08	0.16	0.09	0.07	0.11
*ρ*	−0.53	−0.47	−0.59	−0.70	−0.80	−0.60	−0.03	−0.17	0.11

$\sigma_{a}^{2}\left(\sigma_{a*}^{2}\right)$, additive variance at the level of the mean (variance); $\sigma_{c}^{2}\left(\sigma_{c*}^{2}\right)$, permanent environmental variance at the level of the mean (variance).

## Discussion

This study combined data from 1131, 216 and 221 full‐sib families of GIFT reared in Malaysia, China and India respectively, with family sizes exceeding 32 individuals (Table 1). Sae‐Lim *et al*. (2015b) recommended that, for moderately heritable traits (*h* ^2^ = 0.3), i.e. growth, the optimal data for investigating G × E and micro‐environmental sensitivity consists of at least 100 full‐sib families, each with at least 10 individuals for the G × E and 39 individuals for micro‐environmental sensitivity. Therefore, the data for this study were highly suitable for investigating G × E and micro‐environmental sensitivity.

### G × E

The presence of G × E indicates re‐ranking of breeding values of genotypes across environments. Hence, selection in one environment may not lead to the same expected genetic gain in other production environments (Mulder & Bijma 2005). Practically, genotype re‐ranking should be considered in breeding programmes when genetic correlation is below 0.8 (Robertson 1959; Mulder & Bijma 2005). The presence of the identified moderate to severe G × E in the studied GIFT populations may thus lead to re‐ranking of genotypes across countries in regards to the genetic merit for growth, which may reduce the genetic gain and decrease the efficiency of selection (Mulder *et al*. 2006).

In line with this study, Sae‐Lim *et al*. (2013) found strong G × E in Rainbow Trout reared in different countries. However, previous studies reported weak G × E in GIFT (e.g. Khaw *et al*. 2012). A fundamental difference between ours and most previous G × E studies on GIFT is that, in the latter studies, the environment was usually changed after full‐sibs had been reared in the same hapas for a few months post hatching. In contrast, in the present study, differences in the environment occurred from birth, thus affecting the entire rather than only the latter part of the developmental stages of the fish. To the best of our knowledge, no empirical study to date has monitored the evolution of G × E caused by selection in different environments over time. Our study revealed that genetic correlations can indeed change over successive generations. However, without additional data (e.g. genomic information or environmental parameters such as water temperature or photo‐period over time), determining the causative factors for the observed G × E patterns would only be speculative.

### Micro environmental sensitivity

The genetically structured environmental variance model assumes that there are genes controlling not only the mean of a trait but also its variance (San Cristobal‐Gaudy *et al*. 1998). Our results show the presence of a considerable additive genetic variance for both the mean transformed BW ($\sigma_{a}^{2}$) and its variance ($\sigma_{a^{*}}^{2}$) in GIFT. Evidence for genetic variation in micro‐environmental sensitivity was found in the BW of GIFT (Khaw *et al*. 2012; Marjanovic *et al*. 2016), Rainbow Trout (Janhunen *et al*. 2012; Sae‐Lim *et al*. 2015a) and Atlantic salmon (Sonesson *et al*. 2013). The presence of genetic variation in the residual variance of BW implies that the residual variance can be modified by selection. This can be quantified using the formula of Sonesson *et al*. (2013), $\sigma_{e^{\mathrm{new}}}^{2}=\sigma_{e^{\mathrm{old}}}^{2}*\exp\left(\Delta G_{v}\right)$, where $\sigma_{e^{\mathrm{new}}}^{2}$ is the residual variance after selection, $\sigma_{e^{\mathrm{old}}}^{2}$ is the residual variance prior to selection and Δ*G*_*v*_ is the genetic gain on the underlying log scale of the variance, given by the genetic standard deviation *σ*_a*_. The genetic standard deviation of the residual variance indicates the proportional change in residual variance when increasing/decreasing the residual variance breeding value by one standard deviation unit. For the GIFT populations in this study, the genetic deviations in the variance models of Malaysia, India and China were 0.58, 0.55 and 0.34 respectively. Hence, a unit standard deviation decrease of the breeding value for variance would decrease the residual variance by 44% [exp(−0.58) = 0.56] in Malaysia and by 42% in China, whereas in India the corresponding decrease would be 30%. The genetic standard deviation of the residual variance is valuable because it can be used to compare the results of different experiments and species, as it is not dependent on the phenotypic variance (Mulder *et al*. 2007). In line with our results, Sonesson *et al*. (2013) found an equivalent decrease by 36% for the residual variance of harvest body weight in Atlantic salmon.

Using the Bayesian gsevm v.2 software has an advantage over non‐Bayesian methods of considering common maternal environmental effects; however, due to technical issues, it cannot incorporate a covariance for common environmental effects on mean and variance. Furthermore, the gsevm v.2 software incorporates an animal model to estimate genetic parameters for both mean and variance. Although Bayesian methods are known to deal well with uncertainty in data, the fact that only single observations per animal were available may indeed explain the observed discrepancy in the estimates for the additive genetic variances in mean performance between the models for G × E and micro‐environmental sensitivity (Sorensen & Waagepetersen 2003). This may also have influenced the estimate of the posterior mean of the corresponding genetic correlation (Marjanovic *et al*. 2016). The posterior mean (and 95% credibility interval) of the genetic correlation between the additive genes affecting the mean transformed BW and its variance of GIFT reared in Malaysia and China were moderate and negative in our study. This would indicate that selection for greater (transformed) BW of GIFT would simultaneously increase uniformity in this trait. Marjanovic *et al*. (2016) found different signs, but similar magnitude (0.60 ± 0.09), for the genetic correlations between the additive genes affecting the mean of body weight and its variance using a Box‐Cox transformed dataset of 6090 individuals from the same GIFT population in Malaysia. Data transformations, although common and justified in aquaculture, may affect genetic uniformity parameter estimates (Janhunen *et al*. 2012; Marjanovic *et al*. 2016). Different signs of the genetic correlation estimates were found when using original and transformed body weight data in Rainbow Trout (Sae‐Lim *et al*. 2015a) and in Atlantic salmon (Sonesson *et al*. 2013). Thus, it cannot be excluded that uniformity parameter estimates in our study may partly depend on the chosen square root BW transformation. Nevertheless, the latter was deemed as most appropriate, not only because it best satisfied the normality assumptions of the statistical models but also because it facilitates direct comparison with the G × E analysis results.

### Implications for breeding

Improving the efficiency of breeding programmes for better farmed fish performance across multiple environments is crucial in a globalized aquaculture market. Our results indicate a potential high re‐ranking of breeding candidates in GIFT. These results have important implications for global breeding programmes for tilapia and other species. The presence of G × E means that the selection programme in Malaysia may not be effective in producing fish that also grow faster in other countries. Several strategies can be used to reduce the consequences of the presence of the G × E such as (i) identifying and modifying the environmental conditions of the rearing environments, (ii) running sib evaluations in all environments or (iii) dividing the breeding programme into several environment‐specific breeding programmes (Mulder *et al*. 2006). For GIFT, identifying the environmental variables causing G × E and modifying the rearing environment to be similar to the nucleus is expensive and not feasible. However, collecting sib performance records in the rearing environments could be an option, as this can be used to calculate environment‐specific breeding values. Dividing a single breeding programme of GIFT into several environment‐specific breeding programmes could be a viable alternative. However, developing a separate breeding programme of GIFT for each environment requires large investment and high running costs and, therefore, should be based on a complete cost–benefit study. The identified additive genetic effects controlling the environmental variance of GIFT body weights in different environments creates an opportunity for reducing variation among individuals by selection and thus improving uniformity in harvest weight, which would ease the grading and processing of fish (Mulder *et al*. 2008). Selection for reducing residual variation has already proved to be successful in rabbits, where it led to more homogeneous litters (Garreau *et al*. 2008).

Finally, it would be of considerable value to extend the single‐trait genetic micro‐environmental sensitivity models to multi‐trait models in order to combine the G × E and micro‐environmental sensitivity aspects of robustness into one breeding goal. Selection for this multi‐faceted robustness may improve animal performance and resilience to local environmental fluctuations in different production environments simultaneously.

## Conclusion

Strong G × E was found in the BW of GIFT reared in three different countries: Malaysia, India and China. The environmental variance of BW in GIFT is partly genetically determined. Integrating both G × E and micro‐environmental sensitivity information may help to select robust genotypes with high performance across environments and resilience to environmental fluctuations. Implementing robustness into the breeding objective could be useful in improving multi‐environmental breeding programmes.

## References

[age12680-bib-0001] Falconer D.S. & Mackay T.F.C. (1996) Introduction to Quantitative Genetics. Longman, Harlow, UK.

[age12680-bib-0002] Garreau H. , Bolet G. , Larzul C. , Robert‐Granie C. , Saleil G. , SanCristobal M. & Bodin L. (2008) Results of four generations of a canalising selection for rabbit birth weight. Livestock Science 119, 55–62.

[age12680-bib-0003] Gelman A. & Rubin D.B. (1992) Inference from iterative simulation using multiple sequences. Statistical Science 7, 457–511.

[age12680-bib-0004] Hamzah A. , Ponzoni R.W. , Nguyen N.H. , Khaw H.L. , Hoong Y. & Nor S. (2014) Performance of the Genetically Improved Farmed Tilapia (GIFT) strain over ten generations of selection in Malaysia. Pertanika Journal of Tropical Agricultural Science 374, 411–29.

[age12680-bib-0005] Henderson C.R. (1975) Best linear unbiased estimation and prediction under a selection model. Biometrics 31, 423–47. 1174616

[age12680-bib-0006] Hill W.G. & Mulder H.A. (2010) Genetic analysis of environmental variation. Genetics Research, 92, 381–95. 21429270 10.1017/S0016672310000546

[age12680-bib-0007] Ibanez‐Escriche N. , Varona L. , Sorensen D. & Noguera J.L. (2008) A study of heterogeneity of environmental variance for slaughter weight in pigs. Animal 2, 9–26. 22444959 10.1017/S1751731107001000

[age12680-bib-0008] Ibanez‐Escriche N. , Garcia M. & Sorensen D. (2010) gsevm v.2: MCMC software to analyze genetically structured environmental variance models. Journal of Animal Breeding and Genetics 127, 249–51. 20536643 10.1111/j.1439-0388.2009.00846.x

[age12680-bib-0009] Janhunen M. , Kause A. , Vehvilainen H. & Jarvisalo O. (2012) Genetics of micro‐environmental sensitivity of body weight in Rainbow Trout (*Oncorhynchus mykiss*) selected for improved growth. PLoS ONE 7, e38766. 22701708 10.1371/journal.pone.0038766 PMC3372501

[age12680-bib-0010] Khaw H.L. , Ponzoni R.W. , Hamzah A. , Abu‐Bakar K.R. & Bijma P. (2012) Genotype by production environment interaction in the GIFT strain of Nile tilapia (*Oreochromis niloticus*). Aquaculture 326, 53–60.

[age12680-bib-0011] Knap P.W. (2005) Breeding robust pigs. Australian Journal of Experimental Agriculture 45, 763–73.

[age12680-bib-0012] Marjanovic J. , Mulder H. , Khaw H.L. & Bijma P. (2016) Genetic parameters for uniformity of harvest weight and body size traits in the GIFT strain of Nile tilapia. Genetics Selection Evolution 48, 41. 10.1186/s12711-016-0218-9 PMC4901462 27286860

[age12680-bib-0013] Misztal I. , Tsuruta S. , Lourenco D. , Aguilar I. , Legarra A. & Vitezica Z. (2015). Manual for blupf90 Family of Programs. University of Georgia, AL. http://nce.ads.uga.edu/wiki/lib/exe/fetch.php?media=blupf90_all2.pdf.

[age12680-bib-0014] Mulder H.A. & Bijma P. (2005) Effects of genotype × environment interaction on genetic gain in breeding programs. Journal of Animal Science 83, 49–61. 15583042 10.2527/2005.83149x

[age12680-bib-0015] Mulder H.A. , Veerkamp R.F. , Ducro B.J. , van Arendonk J.A.M. & Bijma P. (2006) Optimization of dairy cattle breeding programs for different environments with genotype by environment interaction. Journal of Dairy Science 89, 1740–52. 16606745 10.3168/jds.S0022-0302(06)72242-1

[age12680-bib-0016] Mulder H.A. , Bijma P. & Hill W.G. (2007) Prediction of breeding values and selection responses with genetic heterogeneity of environmental variance. Genetics 175, 1895–910. 17277375 10.1534/genetics.106.063743 PMC1855112

[age12680-bib-0017] Mulder H.A. , Bijma P. & Hill W.G. (2008) Selection for uniformity in livestock by exploiting genetic heterogeneity of residual variance. Genetics Selection Evolution 40, 37–60. 10.1186/1297-9686-40-1-37 PMC2674918 18096114

[age12680-bib-0018] Mulder H.A. , Ronnegard L. , Fikse W. , Veerkamp R. & Strandberg E. (2013) Estimation of genetic variance for macro‐ and micro‐environmental sensitivity using double hierarchical generalized linear models. Genetics Selection Evolution 45, 23. 10.1186/1297-9686-45-23 PMC3734065 23827014

[age12680-bib-0019] Ponzoni R.W. , Nguyen N.H. , Khaw H.L. , Hamzah A. , Bakar K.R.A. & Yee H.Y. (2011) Genetic improvement of Nile tilapia (*Oreochromis niloticus*) with special reference to the work conducted by the WorldFish Center with the GIFT strain. Reviews in Aquaculture 3, 27–41.

[age12680-bib-0020] Robertson A. (1959) The sampling variance of the genetic correlation coefficient. Biometrics 15, 469–85.

[age12680-bib-0021] Sae‐Lim P. , Kause A. , Janhunen M. , Vehviläinen H. , Koskinen H. , Gjerde B. , Lillehammer M. & Mulder H. (2015a) Genetic (co)variance of Rainbow Trout (*Oncorhynchus mykiss*) body weight and its uniformity across production environments. Genetics Selection Evolution 47, 46. 10.1186/s12711-015-0122-8 PMC4435928 25986847

[age12680-bib-0022] Sae‐Lim P. , Gjerde B. , Marie Nielsen H. , Mulder H. & Kause A. (2015b) A review of genotype‐by‐environment interaction and micro‐environmental sensitivity in aquaculture species. Reviews in Aquaculture 7, 1–25.

[age12680-bib-0901] Sae‐Lim P. , Kause A. , Mulder H. , Martin K. , Barfoot A. , Parsons J. , Davidson J. , Rexroad C.E.I. , van Arendonk J.A.M. & Komen H. (2013) Genotype‐by‐environment interaction of growth traits in rainbow trout (Oncorhynchus mykiss): a continental scale study. Journal of Animal Science 91, 5572–81. 24085417 10.2527/jas.2012-5949

[age12680-bib-0023] San Cristobal‐Gaudy M. , Elsen J.M. , Bodin L. & Chevalet C. (1998) Prediction of response to a selection for canalisation of a continuous trait in animal breeding. Genetics Selection Evolution 30, 423–51.

[age12680-bib-0024] Sonesson A. , Ødegard J. & Ronnegard L. (2013) Genetic heterogeneity of within‐family variance of body weight in Atlantic salmon (*Salmo salar*). Genetics Selection Evolution 45, 41. 10.1186/1297-9686-45-41 PMC4015029 24134557

[age12680-bib-0025] Sorensen D. & Waagepetersen R. (2003) Normal linear models with genetically structured residual variance heterogeneity: a case study. Genetical Research 82, 207–22. 15134199 10.1017/s0016672303006426

[age12680-bib-0026] Strandberg E. , Felleki M. , Fikse W.F. , Franzen J. , Mulder H.A. , Ronnegard L. , Urioste J.I. & Windig J.J. (2013) Statistical tools to select for robustness and milk quality. Advances in Animal Biosciences, 4, 606–11.

